# Supplementary material for: Hybrid Nanocomposite Platform, Based on Carbon Nanotubes and Poly(Methylene Blue) Redox Polymer Synthesized in Ethaline Deep Eutectic Solvent for Electrochemical Determination of 5-Aminosalicylic Acid
Source: Sensors (Basel). 2021 Feb 7;21(4):1161. doi: 10.3390/s21041161 (PMC7915580; doi:10.3390/s21041161)
Supplement: Supplementary file 1 [file sensors-21-01161-s001.pdf]

sensors

MDPI

Supplementary information

Hybrid nanocomposite platform based on carbon nanotubes and poly(methylene blue) redox polymer synthesized in ethaline deep eutectic solvent for electrochemical determination of 5-aminosalicylic acid

Oana Hosu[1,2], Madalina M. Barsan[1,3], Robert Săndulescu[2], Cecilia Cristea[2*], Christopher M.A. Brett[1]

[1] Department of Chemistry, CEMMPRE, Faculty of Sciences and Technology, University of Coimbra, 3004-535 Coimbra, Portugal; hosuoanaalexandra@gmail.com (O.H.); madalina.barsan@gmail.com (M.M.B.); cbrett@ci.uc.pt (C.M.A.B.);

[2] Department of Analytical Chemistry, Faculty of Pharmacy, "Iuliu Haţieganu" University of Medicine and Pharmacy, 400349 Cluj-Napoca, Romania; hosuoanaalexandra@gmail.com; rsandulescu@umfcluj.ro (R.S.); ccristea@umfcluj.ro (C.C.);

[3] National Institute of Material Physics, 077125 Magurele, Romania; madalina.barsan@gmail.com;

* Correspondence: ccristea@umfcluj.ro;

Received: 12 January 2021; Accepted: 4 February 2021; Published: 7 February 2021

There is also an effect of the pH on the electroactive PMB polymer itself, $\Delta E/\Delta$pH plots were constructed based on the anodic peak of the PMB_{DES} platform in BR buffer solutions (Figure S1a) and in the presence of 50 μM 5-ASA (Figure S1b). The oxidation peak potential, E_{pa}, shifts linearly in a negative direction according to: E_{pa} / mV = 267 – 49.0 pH (R^2 = 0.9977), meaning that the same number of protons and electrons are involved in the PMB redox process [1]. 5-ASA does not influence the electrochemical process of PMB as the peak potentials shift negatively by the same amount as for 5-ASA on increasing the pH (Figure S1b).

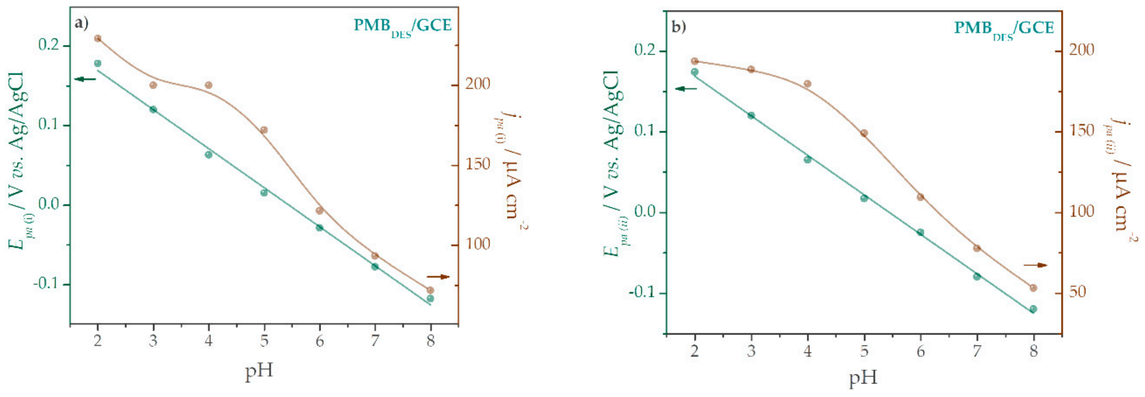

Figure S1. Effect of pH on PMB_{DES} anodic peak current, $j_{pa(i/ii)}$ (μA cm^{-2}, ●) and peak potential, $E_{pa(i/ii)}$ (V, ●) at PMBDES/GCE **a)** in BR buffer solution and **b)** in the presence of 50.0 μM 5-ASA.

An overview of the CA response of 5-ASA at different pH values and applied potentials can be seen in Figure S2a. The influence of pH and applied potential was also evaluated at sensors based on CNT together with PMBDES (Figure S2b). Calibration plots at CNT/PMBDES/GCE in the electrochemical response of 5-ASA at the hybrid composite platform can be seen, probably due to the change in the electrostatic interaction between the -COOH groups present at the CNT surface and 5-ASA at different pH values. PMBDES/GCE showed maximum sensitivity at pH 7.0 at + 0.60 V, the hybrid composite platform CNT/PMBDES/GCE exhibited a sensitivity 4-fold higher than that at + 0.40 V, probably due to electrostatic affinity between the platform and 5-ASA. The sensitivity increased when lower potential values were applied from 0.77 μA cm^{-2} μM^{-1} at E = + 0.60 V to 1.33 μA cm^{-2} μM^{-1} at E = + 0.40 V. This can be explained by the electrocatalytic effect of the hybrid composite that enables the anodic reaction of 5-ASA to occur at lower potential values as will be further confirmed by differential pulse voltammetry.

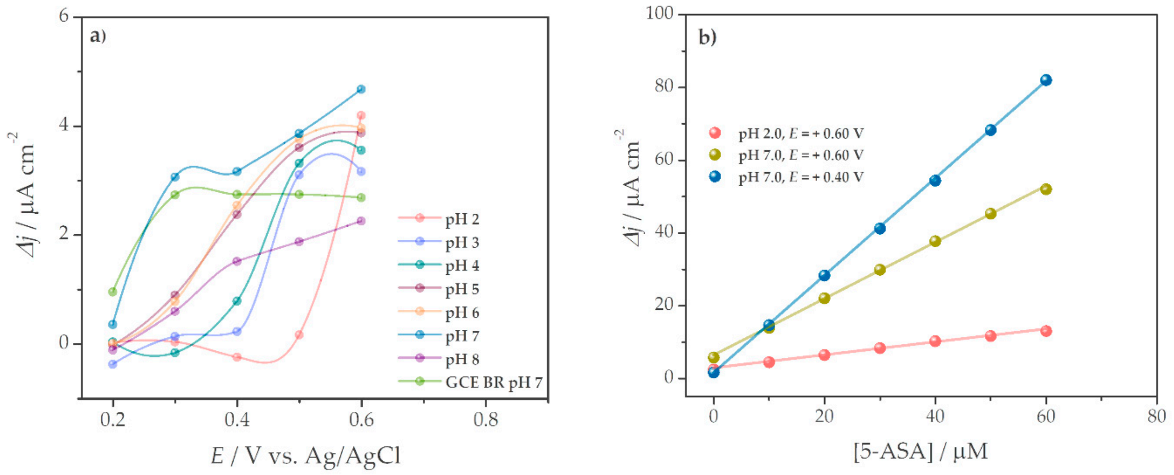

Figure S2. a) The pH effect on CA response of 50 μM 5-ASA at GCE and PMBDES/GCE in 0.04 M BR buffer pH 2.0 – 8.0 at applied potentials between E = + 0.20 V and + 0.60 V; **b)** calibration plots obtained at CNT/PMBDES/GCE in different experimental conditions.

Table S1. Analytical parameters obtained for 5-ASA sensors from corresponding calibration plots (CA in 0.04 M BR pH 7.0, E = + 0.40 V *vs.* Ag/AgCl).

Electrode architecture	Sensitivity / μA cm^{-2} μM^{-1}	RSD* / %	LOD / μM
GCE	0.26	4.2	0.94
PMBDES/GCE	0.35	5.7	0.83
CNT/GCE	0.77	4.1	0.64
PMBDES/CNT/GCE	1.06	2.5	0.19

CNT/PMB$_{DES}$/GCE	1.33	1.6	0.06

*Average of 5 measurements

Reference

1. Brett, C. M. A.; Oliveira-Brett, A.-M. *Electroanalysis*; Compton, R. G., Ed., ; Oxford University Press: New York, **1998**.
